# Tissue Inhibitor of Metalloproteinase-3 Ameliorates Diabetes-Induced Retinal Inflammation

**DOI:** 10.3389/fphys.2021.807747

**Published:** 2022-01-10

**Authors:** Ahmed M. Abu El-Asrar, Ajmal Ahmad, Mohd Imtiaz Nawaz, Mohammad Mairaj Siddiquei, Alexandra De Zutter, Lotte Vanbrabant, Priscilla W. Gikandi, Ghislain Opdenakker, Sofie Struyf

**Affiliations:** ^1^Department of Ophthalmology, College of Medicine, King Saud University, Riyadh, Saudi Arabia; ^2^Dr. Nasser Al-Rashid Research Chair in Ophthalmology, College of Medicine, King Saud University, Riyadh, Saudi Arabia; ^3^Laboratory of Molecular Immunology, Department of Microbiology, Immunology and Transplantation, Rega Institute for Medical Research, KU Leuven, Leuven, Belgium; ^4^Department of Microbiology and Immunology and Transplantation, Rega Institute for Medical Research, University of Leuven, KU Leuven, and University Hospitals UZ Gasthuisberg, Leuven, Belgium

**Keywords:** diabetic retinopathy, tissue inhibitor of metalloproteinase-3, inflammation, angiogenesis, blood-retinal barrier

## Abstract

**Purpose:** Endogenous tissue inhibitor of matrix metalloproteinase-3 (TIMP-3) has powerful regulatory effects on inflammation and angiogenesis. In this study, we investigated the role of TIMP-3 in regulating inflammation in the diabetic retina.

**Methods:** Vitreous samples from patients with proliferative diabetic retinopathy (PDR) and non-diabetic patients were subjected to Western blot analysis. Streptozotocin-treated rats were used as a preclinical diabetic retinopathy (DR) model. Blood-retinal barrier (BRB) breakdown was assessed with fluorescein isothiocyanate (FITC)-conjugated dextran. Rat retinas, human retinal microvascular endothelial cells (HRMECs) and human retinal Müller glial cells were studied by Western blot analysis and ELISA. Adherence of human monocytes to HRMECs was assessed and *in vitro* angiogenesis assays were performed.

**Results:** Tissue inhibitor of matrix metalloproteinase-3 in vitreous samples was largely glycosylated. Intravitreal injection of TIMP-3 attenuated diabetes-induced BRB breakdown. This effect was associated with downregulation of diabetes-induced upregulation of the p65 subunit of NF-κB, intercellular adhesion molecule-1 (ICAM-1), and vascular endothelial growth factor (VEGF), whereas phospho-ERK1/2 levels were not altered. In Müller cell cultures, TIMP-3 significantly attenuated VEGF upregulation induced by high-glucose (HG), the hypoxia mimetic agent cobalt chloride (CoCl_2_) and TNF-α and attenuated MCP-1 upregulation induced by CoCl_2_ and TNF-α, but not by HG. TIMP-3 attenuated HG-induced upregulation of phospho-ERK1/2, caspase-3 and the mature form of ADAM17, but not the levels of the p65 subunit of NF-κB and the proform of ADAM17 in Müller cells. TIMP-3 significantly downregulated TNF-α-induced upregulation of ICAM-1 and VCAM-1 in HRMECs. Accordingly, TIMP-3 significantly decreased spontaneous and TNF-α- and VEGF-induced adherence of monocytes to HRMECs. Finally, TIMP-3 significantly attenuated VEGF-induced migration, chemotaxis and proliferation of HRMECs.

**Conclusion:** *In vitro* and *in vivo* data point to anti-inflammatory and anti-angiogenic effects of TIMP-3 and support further studies for its applications in the treatment of DR.

## Introduction

Diabetic retinopathy (DR) is the most frequent microvascular complication of diabetes mellitus and remains the principal cause of visual impairment among the working-age population. Evidence is accumulating that chronic low-grade subclinical inflammation is fundamental in the initiation and progression of DR (Joussen et al., 2004; Forrester et al., 2020). Enhanced adhesion of circulating leukocytes to the retinal microvascular endothelium actively contributes to the development of retinal endothelial cell damage, breakdown of the blood-retinal barrier (BRB) and capillary non-perfusion (Joussen et al., 2004). The breakdown of the BRB and the concomitant increase in vascular permeability results in diabetic macular edema, which affects vision in diabetic patients (Daruich et al., 2018).

In the ocular microenvironment of patients with PDR, several inflammatory and angiogenic factors are upregulated reinforcing the paradigm that inflammation and angiogenesis are critical mechanisms initiating and supporting progression of PDR (Abu El-Asrar et al., 2019, 2021a,b; Rezzola et al., 2020; Wu et al., 2021). Among these factors, vascular endothelial growth factor (VEGF), released in response to hypoxia, is a key player in promoting retinal angiogenesis and vascular leakage (Peach et al., 2018). VEGF exerts this effect by activating its transmembrane tyrosine kinase-containing receptor VEGFR-2 on vascular endothelial cells (Peach et al., 2018). Despite advances in drug discovery and development, it is still necessary to gain insight into the etiology of DR for allowing for the discovery of novel biomarkers and therapeutic targets. Effective inhibition of diabetes-induced retinal injury might require multiple agents acting on different pathways to attain complete disruption of disease progression.

Tissue inhibitors of metalloproteinases (TIMPs) constitute a family of four members in the human species (TIMP-1, TIMP-2, TIMP-3, and TIMP-4). TIMPs are endogenous inhibitors of matrix metalloproteinases (MMPs) and play critical roles in the maintenance of extracellular matrix (ECM) homeostasis. Although originally identified as inhibitors of MMPs, TIMPs have also been shown to act as multifunctional signaling molecules with cytokine-like activities that are independent of their MMP-inhibitory function (Ries, 2014; Jackson et al., 2017; Eckfeld et al., 2019). TIMP-3 is unique in that, in addition to inhibiting MMPs, TIMP-3 is also an efficient inhibitor of several members of the ADAM (a disintegrin and metalloproteinase), including ADAM17, and ADAMTS (ADAM with thrombospondin motifs) families. As a particular note, ADAM17, also named tumor necrosis factor-α (TNF-α) converting enzyme, activates pro-TNF-α into a key inflammatory mediator. Hence, TIMP-3 possess important signaling functions. TIMP-3 is also distinct from other human TIMPs in that it is sequestered in the ECM (Fan and Kassiri, 2020).

TIMP-3 has emerged as a key mediator limiting inflammation and fibrosis and promoting the resolution of inflammation following injury (Kassiri et al., 2009; Gill et al., 2010, 2013; Fiorentino et al., 2013). TIMP-3 is also a mediator of macrophage polarization and function (Casagrande et al., 2012; Menghini et al., 2012; Gill et al., 2013; Das et al., 2014; Stöhr et al., 2014). In addition to its role in regulating inflammation, several studies demonstrated that TIMP-3 is a potent inhibitor of angiogenesis and suppresses VEGF-mediated angiogenesis independently of its MMP inhibitory properties. Its angiostatic function is mediated by blocking the binding of VEGF to its receptor VEGFR-2 and inhibiting proliferation, migration and tube formation of endothelial cells, key steps in the angiogenesis cascade (Qi et al., 2003, 2013; Chen et al., 2014). Furthermore, in several studies it was reported that TIMP-3 is a potent inhibitor of tumor angiogenesis, growth, inflammatory cell infiltration and metastasis (Spurbeck et al., 2002; Qi et al., 2003; Chen et al., 2014; Das et al., 2014, 2016a,b; Adissu et al., 2015).

In a previous study, we systematically investigated all four human TIMPs in the vitreous fluid from patients with PDR, and we showed that TIMP-1 and TIMP-4 were significantly upregulated in PDR. In contrast, TIMP-2 and TIMP-3 levels were not enhanced in PDR patients, compared to non-diabetic control patients (Abu El-Asrar et al., 2018). We here assessed signaling functions of TIMP-3 *in vitro* and *in vivo* within the context of DR and on the basis of these findings, we hypothesized that enhancing the expression of TIMP-3 could serve as a potential therapeutic strategy for the amelioration of diabetes-induced retinal injury.

## Materials and Methods

### Antibodies and Recombinant Protein

The antibodies used for Western blot analysis in the present study were as follows: rabbit monoclonal anti-phospho-extracellular signal-regulated kinase (ERK)1/2 antibody (1:1,500, MAB1018, R&D Systems, Minneapolis, MA, United States), mouse monoclonal anti-phospho-p65 subunit of nuclear factor-kappa B (NF-κB) antibody (1:500, sc-136548, Santa Cruz Biotechnology Inc., Santa Cruz, CA, United States), rabbit polyclonal anti-phospho vascular endothelial growth factor receptor (VEGFR-2) antibody (1:1,000, ab194806, Abcam, Cambridge, United Kingdom), mouse monoclonal anti-intercellular adhesion molecule-1 (ICAM-1) antibody (1:1,000, sc-8439, Santa Cruz Biotechnology Inc.), mouse monoclonal anti-vascular cell adhesion protein-1 (VCAM-1) antibody (1:1,000, sc-13160, Santa Cruz Biotechnology Inc.), mouse monoclonal anti-VEGF antibody (1:1,500, MAB293, R&D Systems), rabbit polyclonal caspase-3 antibody (1:1,000, sc-7148, Santa Cruz Biotechnology Inc.), rabbit polyclonal ADAM-17 antibody (1:1,000, ab39163, Abcam), and rabbit monoclonal anti-TIMP-3 antibody (1:1,000, ab277794, Abcam). The recombinant proteins used in the present study were as follows: human TIMP-3 (Cat No 973-TM-010, R&D Systems), human tumor necrosis factor-alpha (TNF-α) (Cat No 210-TA, R&D Systems), and human VEGF (Cat No 293-VE-050, R&D Systems).

### Vitreous Samples

Undiluted vitreous fluid samples (0.3–0.6 ml) were obtained from 12 patients with PDR during pars plana vitrectomy, for the treatment of tractional retinal detachment, and/or non-clearing vitreous hemorrhage. We processed these samples as described previously (Abu El-Asrar et al., 2019, 2021a,b) and compared samples from diabetic patients with those of a clinical control cohort. The control group consisted of 12 patients who had undergone vitrectomy for the treatment of rhegmatogenous retinal detachment with no proliferative vitreoretinopathy (PVR). Control subjects were clinically checked to be free from diabetes or other systemic disease.

The study was conducted according to the tenets of the Declaration of Helsinki. All the patients were candidates for vitrectomy as a surgical procedure. All patients signed a preoperative informed written consent and approved the use of the excised vitreous fluid for further analysis and clinical research. The study design and the protocol were approved by the Research Centre and Institutional Review Board of the College of Medicine, King Saud University.

### Diabetic Retinopathy Animal Model

All procedures with animals were performed in accordance with the Association for Research in Vision and Ophthalmology (ARVO) statement for use of animals in ophthalmic and vision research and were approved by the institutional Animal Care and Use Committee of the College of Pharmacy, King Saud University.

Induction of streptozotocin-induced diabetes in rats was done as follows: Adult male Sprague Dawley rats of 8–9 weeks of age (around 200–220 g body weight) were overnight fasted and a single bolus dose of streptozotocin (STZ) (55 mg/kg) in 10 mM sodium citrate buffer, pH 4.5 (Sigma, St. Louis, MO, United States) was injected intraperitoneally. Equal volumes of citrate buffer were injected in age-matched control rats. Seventy-twohours after STZ injection, rats were checked and considered diabetic if their blood glucose levels were in excess of 250 mg/dl. Only diabetic animals were kept under deep anesthesia, and 350 μM of recombinant human TIMP-3 in 5 μl sterilized solution was injected into the vitreous of the right eye. The left eye received an equal volume of sterile phosphate-buffered saline (PBS) as a control. The animals were euthanized 2 weeks after TIMP-3 injection and the retinas were processed for Western blot analysis (Abu El-Asrar et al., 2021b) to assess the effect of TIMP-3 on early inflammatory marker expression.

The effect of TIMP-3 administration on diabetes-induced breakdown of the BRB was evaluated at a later time point. Ten weeks after induction of diabetes, 350 *μ*M of TIMP-3 in 5 *μ*l sterilized solution was injected into the vitreous of the right eye. The left eye received an equal volume of sterile PBS as a control. Retinas were analyzed for BRB breakdown 2 weeks after intravitreal injection of TIMP-3 using FITC-conjugated dextran as previously described (Abu El-Asrar et al., 2019, 2021b). BRB-breakdown was calculated using the following equation, with the results being expressed in μl/g/h.


$$\frac{\mathrm{Retinal}\,\mathrm{FITC}\,\text{-dextran}\,\left(\mu \,g\right)/\mathrm{retinal}\,\mathrm{weight}\,\left(\text{g}\right)}{\mathrm{Plasma}\,\mathrm{FITC}\,\text{-}\,\mathrm{dextran}\,\mathrm{concentration}\,\left(\mu \,g/\mu \,l\right)*\mathrm{circulation}\,\mathrm{time}\,\left(h\right)}$$

### Human Retinal Müller Glial Cell and Human Retinal Microvascular Endothelial Cell Cultures

To corroborate the findings *in vitro* at the level of critical cell types, we used human retinal microvascular endothelial cells (HRMECs) and human retinal Müller glial cells, two major cell types which actively participate in diabetes-induced inflammatory reactions in the retina. Human retinal Müller glial cells (MIO-M1) (a generous gift from Prof. A. Limb, Institute of Ophthalmology, University College London, United Kingdom) and HRMECs (Cell Systems Corporation, Kirkland, WA, United States) were cultured as described previously (Abu El-Asrar et al., 2019, 2021a,b). Müller cell cultures were either left untreated or stimulated for 24 h. The following stimuli were used: 300 μM of the hypoxia mimetic agent cobalt chloride (CoCl_2_) (Cat No A1425-L, Avonchem Limited, United Kingdom), 25 mM glucose (Cat No GL0125100, Scharlau S.L, Gato Prez, Spain), or 50 ng/ml recombinant human TNF-α in the absence or presence of 1 h pretreatment with TIMP-3 (100 ng/ml). For high-glucose (HG) treatment, 25 mM mannitol (Cat No MA01490500, Scharlau S.L, Gato Prez, Spain) was used as a control. HRMECs were treated with 50 ng/ml recombinant human VEGF or 50 ng/ml recombinant human TNF-α for 24 h in the absence or presence of 1 h pretreatment with human TIMP-3 (100 ng/ml). Cell supernatants were collected for ELISA analysis. Cells were lysed in radioimmunoprecipitation assay (RIPA) lysis buffer; sc-24948, Santa Cruz Biotechnology, Inc., Santa Cruz, CA, United States) for Western blot analysis.

### Western Blot Analysis

Rat retina, cell lysates and vitreous samples were analyzed. Incubation with primary and secondary antibodies was carried out as described previously (Abu El-Asrar et al., 2019, 2021a,b). To verify similar sample loading, membranes were stripped and reprobed with β-actin-specific antibody (1:3,000, sc-47778, Santa Cruz Biotechnology Inc.). Bands were visualized with the use of high-performance chemiluminescence (G: Box Chemi-XX8 from Syngene, Synoptic Ltd., Cambridge, United Kingdom) and the band intensities were quantified with the use of GeneTools software (Syngene by Synoptic Ltd.).

### Enzyme-Linked Immunosorbent Assays

Enzyme-linked immunosorbent assay (ELISA) kits for human monocyte chemotactic protein (MCP-1)/CCL2 (Cat No DCP00), and human VEGF (Cat No DY293B) were purchased from R&D Systems. Levels of human MCP-1/CCL2 and VEGF in culture medium were determined with the aforementioned ELISA kits according to the manufacturer’s instructions. The minimum detection limits for MCP-1/CCL2 and VEGF ELISA kits were 10 and 31.2 pg/ml, respectively.

### Monocyte-Endothelial Cell Adhesion Assay

Monocyte-endothelial cell adhesion was assessed using CytoSelect Leukocyte-endothelium adhesion kit (Cat. No. CBA-210, Cell Biolabs, Inc., San Diego, CA, United States) following the assay protocol provided by the supplier. Briefly, 2 × 10^5^ HRMECs were seeded on 0.2% (v/v) gelatin-coated 24-well plates (Abu El-Asrar et al., 2021a). After reaching a confluent monolayer and overnight starvation, cells were stimulated with 25 ng/ml recombinant human TNF-α or 50 ng/ml recombinant human VEGF for 24 h with or without a 1-h pretreatment with 100 ng/ml TIMP-3. To investigate the capacity of TIMP-3 to inhibit the basal binding of THP-1 monocytes (American Type Culture Collection, Manassas, VA, United States) to HRMECs, overnight starved THP-1 monocytes were treated with or without 100 ng/ml recombinant human TIMP-3 for 24 h.

Next, 5 × 10^5^ fluorescent-LeukoTracker labeled monocytic THP-1 cells were added to the HRMEC monolayer for 60 min. After washing, the remaining adherent THP-1 cells were lysed and fluorescence was measured using a SpectraMax Gemini-XPS (Molecular Devices, CA, United States) with excitation and emission wavelengths of 485 and 538 nm, respectively.

### *In vitro* Angiogenesis Assays

Human Retinal microvascular endothelial cells were seeded at 1 × 10^5^ cells/well on 6-well culture plates and allowed to grow till 80–90% confluency. Quiescence was induced by incubating the cells overnight in minimal medium. Using sterile pipette tips, scratches were made in the monolayers, and detached cells were removed with PBS. Next, part of the wells were incubated with 100 ng/ml TIMP-3 for 1 h and subsequently stimulated with 50 ng/ml recombinant VEGF for 24 h. To corresponding control wells, only minimal medium was added. Cell migration was monitored using an inverted microscope (Olympus IX81, Olympus Corporation, Tokyo, Japan). Analysis of migration was done using Image J software.

Chemotaxis of HRMECs was evaluated using an xCelligence apparatus [Real Time Cell Analyzer-Double Plate (RTCA-DP) system; ACEA Biosciences, Inc., San Diego, CA, United States]. First, the lower chamber of a Cell Invasion/Migration (CIM)-Plate (ACEA Biosciences, Inc) was loaded with 10 ng/ml VEGF or dilution medium [MCDB131 medium (Gibco, Thermo Fisher Scientific, Merelbeke, Belgium) supplemented with 0.4% (v/v) fetal calf serum (FCS)]. Subsequently, the upper part of the chamber was mounted on top of the bottom plate and 50 μL of serum-free MCDB131 medium (pure or containing 10 or 100 ng/ml of TIMP-3) was added to the top wells. After equilibration for 1 h at 37°C, 4 × 10^4^ HRMECs were added to the top wells (100 μL/well) that underwent a 30-min pre-incubation with serum-free MCDB131 medium, or TIMP-3 (10 or 100 ng/ml). Migration was monitored in the RTCA-DP system after an additional incubation (30 min, room temperature), allowing the cells to settle onto the membrane. The rate of chemotaxis, recorded as changes in electrical impedance, was monitored every minute for 15 h. In total five experiments were performed and conditions were tested in duplicate or triplicate within 1 experiment.

To assess the influence of TIMP-3 on the proliferative effect of VEGF, HRMECs were seeded in a 96-well plate (5 × 10^3^ cells in 100 μl/well) in Endothelial Cell Basal Medium-2 (EBM-2) supplemented with the SingleQuots kit (both Lonza, Verviers, Belgium). The next day, cells were washed with serum-free MCDB131 medium and starved in serum-free MCDB medium, supplemented with 2 mM GlutaMAX and 30 μg/ml Gentamicin (Gibco) for 4 h at 37°C, 5% CO_2_. After starvation, cells were preincubated with 0, 10, or 100 ng/mL TIMP-3 in MCDB131 medium, supplemented with 2 mM GlutaMAX, 30 μg/ml Gentamicin and 1% FCS (proliferation medium) for 30 min at 37°C, 5% CO_2_. Finally, cells were stimulated with 10 ng/ml VEGF in proliferation medium or proliferation medium only. After 72 h, cell proliferation was measured using the ATPlite Luminescence Assay kit (Perkin Elmer, Waltham, MA, United States) according to the manufacturer’s instructions.

### Statistical Analysis

Data were collected, stored and managed in a spreadsheet using Microsoft Excel 2010^^®^^ software. Data were analyzed and figures prepared using SPSS^^®^^ version 21.0 (IBM Inc., Chicago, IL, United States). Tests for normality were done using Shapiro-Wilk test and Q-Q plots. The normally distributed data were presented using bar charts showing the standard deviations, while the not normally distributed data were presented using box and whisker plots showing the medians, upper and lower quartiles and range. Consequently, one-way ANOVA and independent *t*-test or Kruskal-Wallis and Mann Whitney tests (applying Bonferroni correction where necessary) were used to test the differences between the groups for normally distributed data and non-normally distributed data, respectively. Any output with a *p* below 0.05 was interpreted as an indicator of statistical significance.

## Results

### The Glycosylated Form of Tissue Inhibitor of Matrix Metalloproteinase-3 Is Upregulated in Vitreous Samples From Patients With Proliferative Diabetic Retinopathy

**FIGURE 1:**
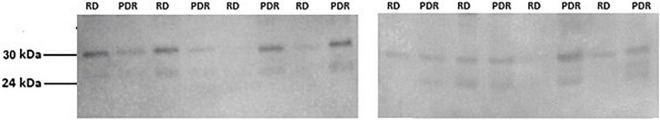
Expression of TIMP-3 in vitreous fluid samples. Equal volumes (15 μl) of vitreous fluid samples from patients with proliferative diabetic retinopathy (PDR) (*n* = 12) and non-diabetic patients with rhegmatogenous retinal detachment (RD) (*n* = 12) were subjected to gel electrophoresis and the presence of TIMP-3 was detected by Western blot analysis. A representative set of samples is shown.

In a previous study, with the use of ELISA, we demonstrated that mean TIMP-3 levels did not differ significantly between PDR patients and non-diabetic control patients (Abu El-Asrar et al., 2018). In the present study, we added Western blot analysis to provide insights into the relative abundance of the various proteoforms and fragments of TIMP-3. With the use of Western blot analysis of equal volumes of vitreous fluid, we confirmed the presence of TIMP-3 in vitreous samples. TIMP-3 immunoreactivities were expressed as two protein bands at approximately 24 and 30 kDa. These correspond by their molecular weights to the previously reported unglycosylated and glycosylated forms of TIMP-3, respectively (Langton et al., 1998; Spurbeck et al., 2002). Most of the TIMP-3 immunoreactivity appeared at the level of 30 kDa form indicating that TIMP-3 in vitreous samples is largely glycosylated (Figure 1).

### Intravitreal Administration of Tissue Inhibitor of Matrix Metalloproteinase-3 Attenuates Diabetes-Induced Breakdown of Blood-Retinal Barrier and Retinal Expression of the p65 Subunit of NF-κB, Intercellular Adhesion Molecule-1, and Vascular Endothelial Growth Factor

**FIGURE 2:**
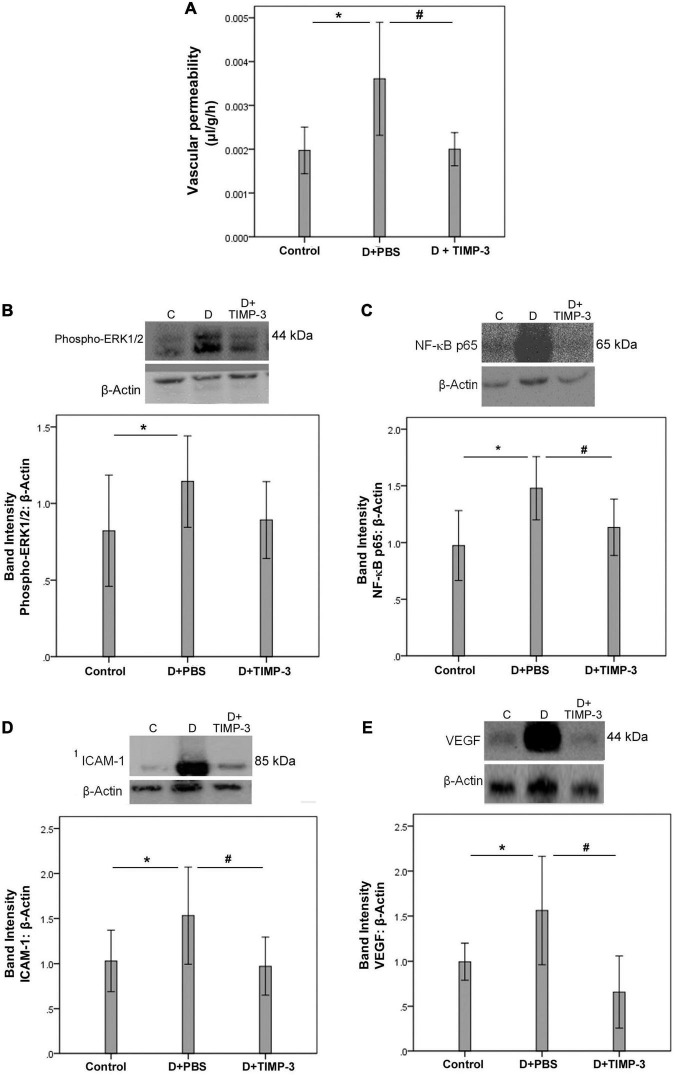
Tissue inhibitor of matrix metalloproteinase-3 (TIMP-3) attenuates retinal inflammation and prevents diabetes-induced blood-retinal barrier (BRB) breakdown. The BRB breakdown [panel **(A)**] was quantified with the fluorescein isothiocyanate-conjugated dextran technique after treatment with intravitreal injection of 350 μM TIMP-3 in 5 μl in one eye and the same volume of phosphate-buffered saline (PBS) in the contralateral eye of rats 10 weeks after induction of diabetes. Results are expressed as mean ± standard deviation of five rats in each group. Evaluation of inflammatory mediators was evaluated shortly after diabetes induction (see section “Materials and Methods”). Western blot analysis of rat retinas was performed to evaluate protein expression levels of phospho-ERK1/2 [panel **(B)**], the p65 subunit of NF-κB [panel **(C)**], intercellular adhesion molecule-1 (ICAM-1) [panel **(D)**], and vascular endothelial growth factor (VEGF) [panel **(E)**]. Results are expressed as mean ± standard deviation of 12 rats in each group. One-way ANOVA and independent *t*-test were used for comparisons between the three and two groups, respectively, panels **(A–E)**. *p < 0.05 compared with non-diabetic controls. ^#^*p* < 0.05 compared with PBS-treated diabetic rats.

With the observed association of increased 30 kDa TIMP-3, and PDR, it was of importance to evaluate whether such increased levels of TIMP-3 are detrimental, beneficial or have no effect *in vivo*. FITC-conjugated dextran was used to investigate the extent of vascular permeability. In STZ-induced diabetic rats, retinal vascular permeability was significantly increased at 12 weeks after the induction of diabetes when compared with non-diabetic rats. Intravitreal treatment with recombinant human TIMP-3 significantly attenuated the diabetes-induced BRB breakdown, compared to PBS-treated diabetic rats (Figure 2A).

Western blot analysis of homogenized retinal tissue revealed that diabetes significantly increased the protein levels of phospho-ERK1/2 (Figure 2B), the p65 subunit of NF-κB (Figure 2C), ICAM-1 (Figure 2D), and VEGF (Figure 2E) at 2 weeks after the induction of diabetes when compared with the retinas of non-diabetic control rats. Treatment with intravitreal TIMP-3 significantly reduced the expression of the p65 subunit of NF-κB (Figure 2C), ICAM-1 (Figure 2D), and VEGF (Figure 2E) proteins in STZ-induced diabetic rats when compared with the values obtained from the PBS-treated contralateral eye. However, TIMP-3 did not affect the expression of phospho-ERK1/2 (Figure 2B).

### Tissue Inhibitor of Matrix Metalloproteinase-3 Attenuates the Expression of Angiogenic and Inflammatory Molecules Induced by Diabetic Mimetic Conditions in Human Retinal Müller Glial Cells

**FIGURE 3:**
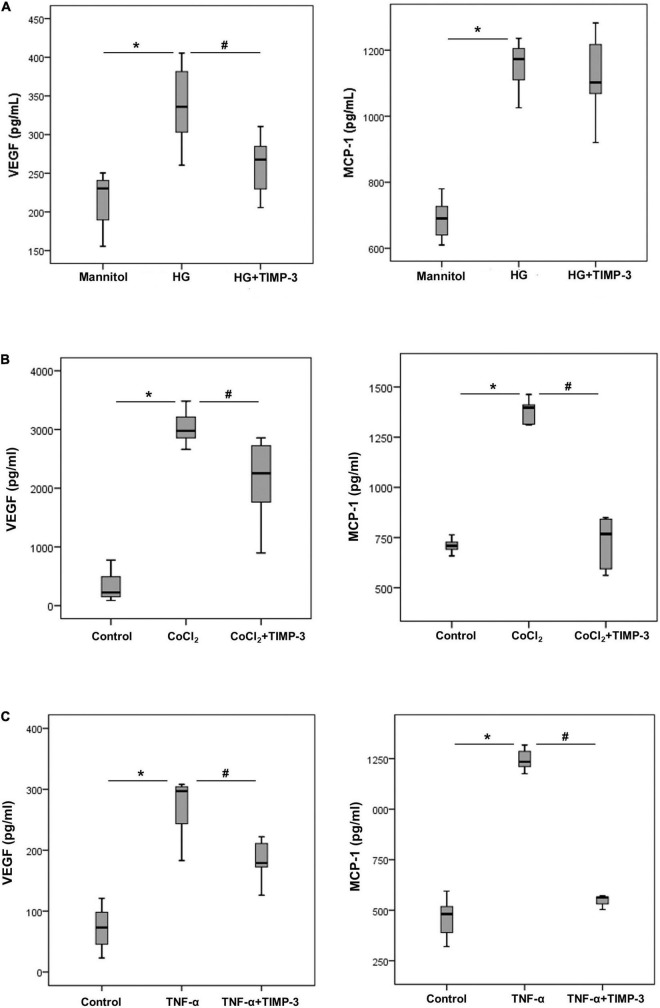
Müller cells were left untreated or treated with high-glucose (HG, 25 mM) [panel **(A)**] cobalt chloride (CoCl_2_) (300 μM) [panel **(B)**] or tumor necrosis factor -α (TNF-α) (50 ng/ml) [panel **(C)**] for 24 h or TIMP-3 (100 ng/ml) for 1 h followed by HG, CoCl_2_, or TNF-α. For HG treatment, cultures containing 25 mM mannitol were used as a control. Levels of vascular endothelial growth factor (VEGF) and monocyte chemotactic protein-1 (MCP-1) were quantified in the culture media by ELISA. Results are expressed as median (interquartile range) from three different experiments performed in triplicate. Kruskal-Wallis test and Mann-Whitney test were used for comparison between three groups and two groups, respectively. *p < 0.05 compared with values obtained from control cells. ^#^*p* < 0.05 compared with values obtained from cells treated with HG, CoCl_2_, or TNF-α.

To better understand the observed alterations *in vivo*, we investigated the molecular effects by TIMP-3 on leukocytes, Müller cells and HRMECs with the use of various assays applying condition relevant in the context of DR. With the use of ELISA analysis, we demonstrated that treatment of Müller cells with the diabetic mimetic conditions HG (Figure 3A), the hypoxia mimetic agent CoCl_2_ (Figure 3B) and the proinflammatory cytokine TNF-α (Figure 3C) induced significant upregulation of the proangiogenic factor VEGF and the inflammatory chemokine MCP-1/CCL2 in the culture medium as compared to untreated controls. Pre-incubation of Müller cells with TIMP-3 significantly attenuated the levels of VEGF induced by HG, CoCl_2_, and TNF-α. TIMP-3 significantly attenuated upregulation of MCP-1/CCL2 induced by CoCl_2_ and TNF-α, but not by HG.

### Tissue Inhibitor of Matrix Metalloproteinase-3 Counteracts High-Glucose-Induced Upregulation of Phospho-ERK1/2, the Apoptosis Executer Enzyme Caspase-3 and ADAM17 in Human Retinal Müller Glial Cells

**FIGURE 4:**
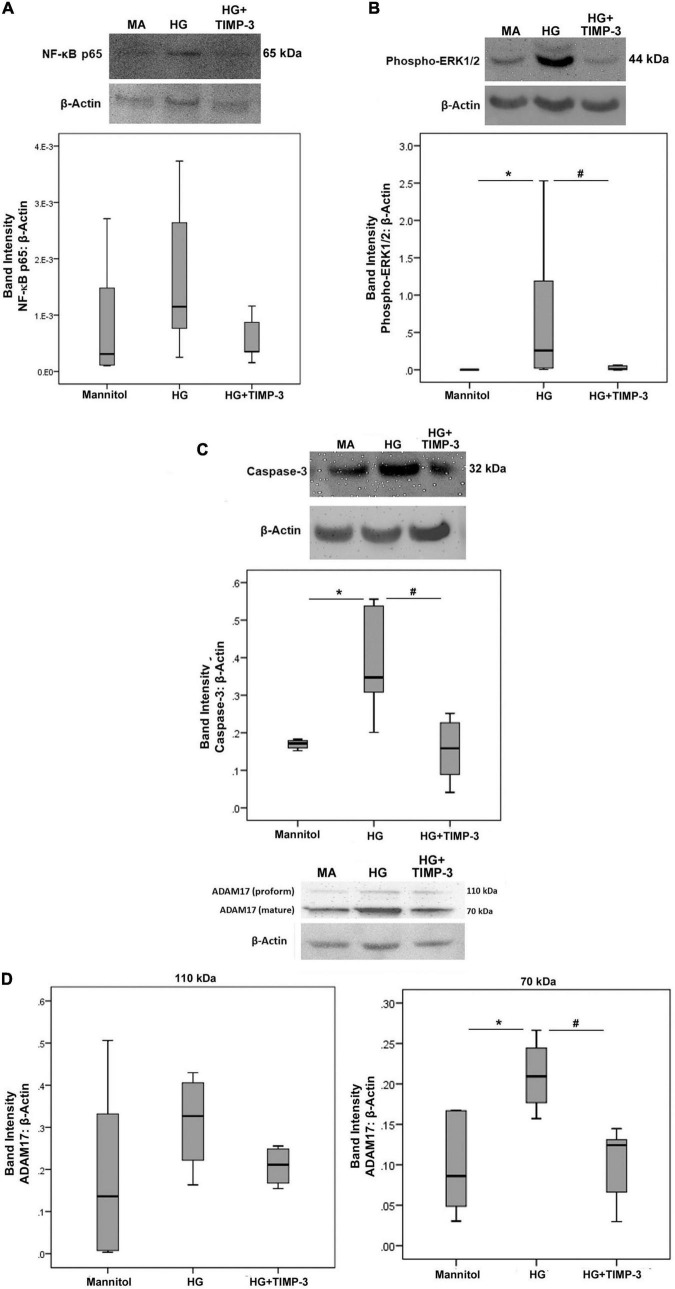
Müller cells were pre-incubated with TIMP-3 (100 ng/ml) or dilution medium for 1 h before increasing the sugar content of the cultures [25 mM of mannitol as control or 25 mM of glucose (HG)]. After 24 h levels of the p65 subunit of NF-κB [panel **(A)**], phospho-ERK1/2 [panel **(B)**], caspase-3 [panel **(C)**], and ADAM17 [panel **(D)**] in cell lysates was determined by Western blot analysis. Results are expressed as median (interquartile range) from three different experiments performed in triplicate. Kruskal-Wallis test and Mann-Whitney test were used for comparisons between three groups and two groups, respectively. *p < 0.05 compared with values obtained from cells treated with mannitol. ^#^*p* < 0.05 compared with values obtained from cells treated with HG.

With the use of Western blot analysis, we demonstrated that treatment of Müller cells with HG induced significant upregulation of the protein levels of phospho-ERK1/2 (Figure 4B), the apoptosis executer enzyme caspase-3 (Figure 4C), and the mature form of ADAM17 (Figure 4D). However, treatment of Müller cells with HG did not affect the expression of the p65 subunit of NF-κB (Figure 4A) and the proform of ADAM17 (Figure 4D). Pretreatment with TIMP-3 significantly attenuated HG-induced upregulation of phospho-ERK1/2 (Figure 4B), caspase-3 (Figure 4C), and the mature form of ADAM17 (Figure 4D). However, TIMP-3 did not affect the expression of the p65 subunit of NF-κB (Figure 4A) and the proform of ADAM17 (Figure 4D).

### Tissue Inhibitor of Matrix Metalloproteinase-3 Reduces THP-1 Cell Adhesion to Human Retinal Microvascular Endothelial Cells

**FIGURE 5:**
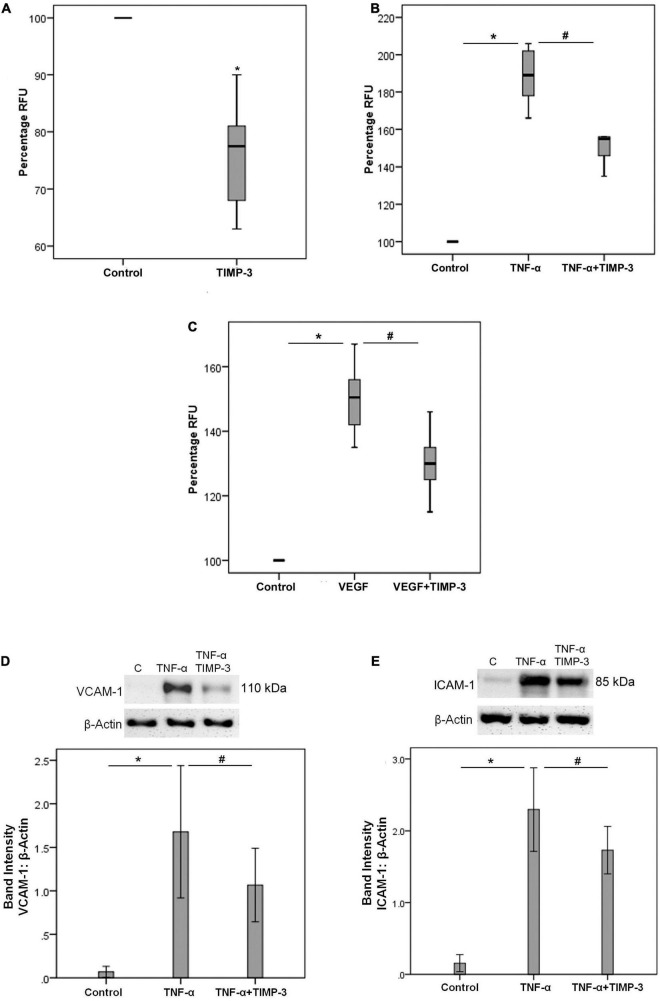
THP-1 monocytes were left untreated or treated with TIMP-3 (100 ng/ml) for 24 h. Subsequently, the THP-1 cells were fluorescently labeled and adhesion to a human retinal microvascular endothelial cell (HRMEC) monolayer was assessed [panel **(A)**]. Results are expressed as median (interquartile range) from two independent experiments (each treatment condition: 6 wells) (*p < 0.05; Mann-Whitney test). Alternatively, HRMECs were pre-incubated with TIMP-3 (100 ng/ml) or dilution medium for 1 h before stimulation with dilution medium, tumor necrosis factor-α (TNF-α) (25 ng/ml) [panel **(B)**] or vascular endothelial growth factor (VEGF) (50 ng/ml) [panel **(C)**] for 24 h. Adhesion of fluorescently labeled monocytic cells to the HRMEC monolayer was assessed. Results are expressed as median (interquartile range) from three independent experiments (each treatment condition: 6 wells). Kruskal-Wallis test and Mann-Whitney test were used for comparisons between three groups and two groups, respectively (RFU = relative fluorescence unit). HRMECs were left untreated or were stimulated with TNF-α (50 ng/ml) for 24 h with/without a 1-h pre-incubation with TIMP-3 (100 ng/ml). Protein expression of vascular cell adhesion molecule-1 (VCAM-1) [panel **(D)**] and intercellular adhesion molecule-1 (ICAM-1) [panel **(E)**] was determined by Western blot analysis. Results are expressed as mean ± standard deviation from three independent experiments (each treatment condition: 8 wells). One-way ANOVA and independent *t*-test were used for comparisons between three groups and two groups, respectively. *p < 0.05 compared with values obtained from untreated cells. ^#^*p* < 0.05 compared with values obtained from cells treated with TNF-α or VEGF.

Increased expression of retinal ICAM-1 and enhanced adhesion of circulating leukocytes to the retinal vascular endothelium are hallmark features of DR (Joussen et al., 2004). We found that treatment of THP-1 cells with TIMP-3 significantly decreased the adherence of monocytes to HRMECs (Figure 5A). In addition, TIMP-3 pretreatment of HRMECs significantly decreased the upregulation of the adherence of monocytes to HRMECs induced by TNF-α (Figure 5B) and VEGF (Figure 5C). Furthermore, TIMP-3 significantly reduced TNF-α-induced upregulation of the adhesion molecules VCAM-1 (Figure 5D) and ICAM-1 (Figure 5E) in HRMECs. These findings suggest that TIMP-3 may protect against inflammatory stimulation in HRMECs during the progression of DR.

### Tissue Inhibitor of Matrix Metalloproteinase-3 Inhibits Vascular Endothelial Growth Factor-Induced Migration, Chemotaxis and Proliferation of Human Retinal Microvascular Endothelial Cells

**FIGURE 6:**
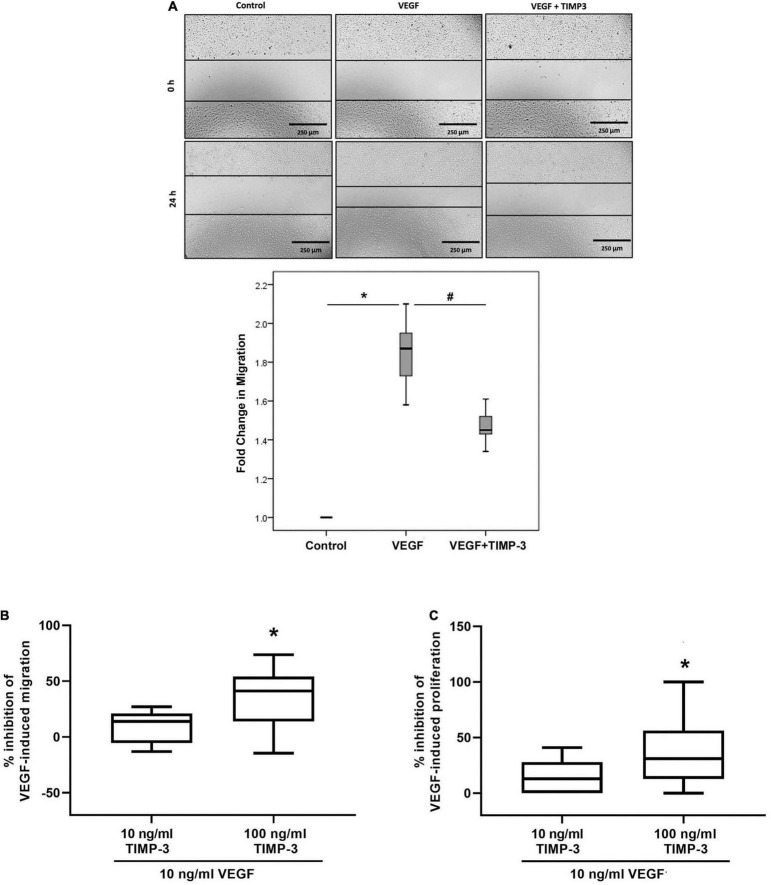
Tissue inhibitor of matrix metalloproteinase-3 (TIMP-3) inhibits vascular endothelial growth factor (VEGF)-mediated human retinal microvascular endothelial cell (HRMEC) migration, chemotaxis and proliferation. A scratch was made in confluent monolayers of overnight starved HRMECs with a micropipette tip. Subsequently, the cultures were pre-incubated with TIMP-3 (100 ng/ml) or dilution medium for 1 h before stimulation with dilution medium or vascular endothelial growth factor (VEGF) (50 ng/ml) for 24 h. Cells were visualized using an inverted microscope. Three independent experiments were performed. Each experiment was done in triplicate and 6–8 independent field images were taken for the migration analysis which was done by using image J software. In the figure one representative image is shown [panel **(A)**]. Results are expressed as median (interquartile range). Kruskal-Wallis test and Mann-Whitney test were used for comparisons between three groups and two groups, respectively. *p < 0.05 compared with untreated cells. ^#^*p* < 0.05 compared with VEGF-treated cells. Chemotaxis and proliferation of HRMECs stimulated with 10 ng/ml VEGF was modulated by a 30-min pre-incubation with TIMP-3 (10 or 100 ng/ml). Cell migration was monitored using the xCELLigence RTCA-DP system. The median (interquartile range) percentage of inhibition of VEGF-induced chemotaxis [panel **(B)**] or proliferation [panel **(C)**] is shown. In total five chemotaxis experiments were performed and conditions were tested in duplicate or triplicate within 1 experiment. For proliferation, 6 experiments were performed and conditions were tested at least in triplicate within 1 experiment. *p < 0.05; Mann-Whitney test (compared with VEGF).

Migration, chemotaxis and proliferation of endothelial cells are critical components of angiogenesis. We tested TIMP-3 for its ability to block migration of HRMECs. TIMP-3 pretreatment significantly attenuated VEGF-induced migration of HRMECs in the scratch wound migration assay (Figure 6A). Similarly, when HRMECs were pretreated with 100 ng/ml TIMP-3, the chemotactic effect of VEGF was inhibited with about 40% (Figure 6B). In contrast, TIMP-3 at 10 ng/ml only marginally inhibited the VEGF-induced migration and the 14% reduction was not statistically significant (Figure 6B). Finally, preincubation of HRMECs with 100 ng/ml TIMP-3 could partially (38%), but significantly inhibit the VEGF-induced proliferation of the endothelial cells (Figure 6C).

## Discussion

In the present study, we demonstrated that local treatment with intravitreal TIMP-3 attenuated the increase in retinal vascular leakage and BRB breakdown in STZ-induced diabetic rats. These findings are in line with previous studies documenting that TIMP-3 preserves blood-brain barrier function in a model of traumatic brain injury (Menge et al., 2012) and attenuates the increase in pulmonary microvascular endothelial cell permeability under septic conditions (Arpino et al., 2016). More recently, Dave et al. (2018) used the same recombinant TIMP-3 material, as used here, to demonstrate *in vivo* that TIMP-3 stabilizes the developing blood-brain barrier and attenuates germinal matrix brain hemorrhage in mice.

The mechanisms by which TIMP-3 attenuated diabetes-induced BRB disruption might be multifold. In this study, we confirmed that diabetes induced a clear upregulation of the retinal expression of VEGF, a key inducer of diabetes-induced breakdown of the BRB (Peach et al., 2018), and we demonstrated that intravitreal TIMP-3 administration normalized retinal VEGF expression. With the use of *in vitro* studies with two critical cell types, Müller cells and retinal endothelial cells, we tried to obtain mechanistic insights into the mechanism(s) of action of TIMP-3. Müller cells are known to be the major source of VEGF secretion in the retina (Bringmann et al., 2006). We demonstrated that TIMP-3 attenuates upregulation of VEGF in human retinal Müller glial cells induced by the hypoxia mimetic agent CoCl_2_, HG or the proinflammatory cytokine TNF-α. We also demonstrated the capability of HG to target Müller cells and to induce activation of the ERK1/2 signaling pathway and that TIMP-3 significantly attenuated the HG-induced upregulation of phospho-ERK1/2. In line with our data, in a previous study it was demonstrated that TIMP-3 inhibited VEGF-stimulated phosphorylation of ERK1/2 in endothelial cells (Qi et al., 2013). In addition, TIMP-3 deficiency increased the levels of phospho-ERK1/2 in the kidney from diabetic mice (Fiorentino et al., 2013). TIMP-3 administration could also attenuate diabetes-induced BRB breakdown through its anti-inflammatory activity. Intravitreal treatment with TIMP-3 attenuated diabetes-induced upregulation of the pro-inflammatory transcription factor NF-κB and the adhesion molecule ICAM-1. Our data also suggest that TIMP-3 has anti-inflammatory effects through the attenuation of VEGF- or TNF-α-stimulated binding of human monocytes to HRMECs. Increased expression of retinal ICAM-1 and enhanced adhesion of circulating leukocytes to the retinal microvascular endothelium are crucial in the development of diabetes-induced retinal endothelial cell damage and breakdown of BRB (Joussen et al., 2004). We also demonstrated that TIMP-3 significantly attenuated TNF-α-induced upregulation of the adhesion molecules ICAM-1 and VCAM-1 in HRMECs. ICAM-1 and VCAM-1 play an important role in promoting leukocyte transmigration (Cook-Mills and Deem, 2005). Additionally, we demonstrated that TIMP-3 significantly attenuated the upregulation of the chemokine MCP-1/CCL2 that promotes migration and recruitment of monocytes (Rana et al., 2018), in Müller cells induced by the hypoxia mimetic agent CoCl_2_ or TNF-α. Moreover, treatment with TIMP-3 effectively attenuated HG-induced upregulation of the proinflammatory factor tumor necrosis factor-α (TNF-α) convertase (TACE/ADAM17) and the apoptosis executer enzyme caspase-3 in Müller cells. Similarly, TIMP-3 overexpression suppressed inflammatory response and apoptosis in HG-treated podocytes (Chen et al., 2018).

These findings are in agreement with previous studies that demonstrated that TIMP-3 is a powerful regulator of inflammation. In mouse models of acute lung injury, TIMP-3 deletion resulted in a markedly elevated and persistent inflammatory response due to a pronounced increase in the number of infiltrated neutrophils and macrophages (Gill et al., 2010, 2013). In a mouse model of unilateral ureteral obstruction, mice lacking TIMP-3 exhibited increased renal injury, increased activation of fibroblasts and greater interstitial fibrosis (Kassiri et al., 2009). Deficiency of TIMP-3 leads to increased macrophage infiltration in the kidney and exacerbates renal damage in response to chronic hyperglycemic stress caused by diabetes (Fiorentino et al., 2013). TIMP-3-deficient tumors, showed markedly increased inflammatory cell infiltration along with increased expression of MCP-1, TNF-α and interleukin-1β (Adissu et al., 2015). In relation to inflammation, TIMP-3 is also a mediator of macrophage polarization and function. In the absence of TIMP-3, macrophage differentiation was altered, resulting in macrophages that were skewed toward a more proinflammatory polarization (Gill et al., 2013). In a mouse model of atherosclerosis, lack of TIMP-3 increases inflammation and polarizes macrophages toward a more inflammatory phenotype resulting in increased atherosclerosis (Stöhr et al., 2014). As a complementation of these data, overexpression of TIMP-3 in macrophages leads to smaller, more stable atherosclerotic plaques that contained fewer inflammatory cells (Casagrande et al., 2012). Similarly, in a mouse model, overexpression of TIMP-3 in macrophages protects from metabolic inflammation and related metabolic disorders such as insulin resistance, glucose intolerance and non-alcoholic steatohepatitis (Menghini et al., 2012).

Angiogenesis, the process by which new capillaries are formed by sprouting from existing vessels, is a fundamental requirement for PDR initiation and progression. VEGF plays a pivotal role in promoting retinal vascular leakage and angiogenesis in DR (Peach et al., 2018). Designing effective therapeutic strategies against PDR-associated angiogenesis requires further understanding of the dynamic balance between proangiogenic and antiangiogenic factors in the ocular microenvironment of patients with PDR. Restoration of this balance between the angiogenic stimulators and inhibitors by activating endogenous angiogenesis inhibitors can become a potential strategy for PDR therapy. Interestingly, in the present study, we demonstrated that the predominant proteoform of TIMP-3 in vitreous samples corresponds to that of glycosylated TIMP-3. Qi et al. (2009) reported that glycosylation lead to a reduction in MMP inhibitory activity of a TIMP-3 mutant with a consequent increase of VEGF-dependent endothelial cell migration and tube formation. In the present study, we demonstrated that TIMP-3 attenuated VEGF-induced HRMECs migration, chemotaxis and proliferation, crucial steps in the angiogenesis cascade. Similarly, several studies demonstrated that TIMP-3 is a potent inhibitor of tumor-associated angiogenesis (Spurbeck et al., 2002; Qi et al., 2003; Chen et al., 2014; Das et al., 2014, 2016a,b; Adissu et al., 2015). In addition, intravitreal injection of TIMP-3 also inhibits oxygen-induced retinal neovascularization (Hewing et al., 2013) and laser-induced choroidal neovascularization (Qi et al., 2013). In addition, TIMP-3 protects against hemorrhage in the developing brain (Dave et al., 2018).

In conclusion, our results demonstrate an important role for TIMP-3 in the pathogenesis of diabetes-induced retinal inflammation. In our study, we used both *in vitro* and *in vivo* models to investigate the anti-inflammatory and anti-angiogenic effects of TIMP-3 in the diabetic retina. Our findings suggest that pharmacological enhancement of local endogenous TIMP-3 levels or local administration of exogenous TIMP-3 proteoforms would be a potential therapeutic strategy which could exert biological effects in several ways. However, more investigations are needed to explore the use of slow release formulations. Despite the progress provided by our study, we have not verified the mechanisms by which TIMP-3 interacts with VEGF and exerts its biological effects. Understanding the mechanisms through which TIMP-3 interferes with VEGF could pave the way for the rational design of drugs that disrupt the progression of diabetes-induced retinal injury.

## Data Availability Statement

The raw data supporting the conclusions of this article will be made available by the authors, without undue reservation.

## Ethics Statement

The studies involving human participants were reviewed and approved by the Research Centre and Institutional Review Board of the College of Medicine, King Saud University. The patients/participants provided their written informed consent to participate in this study. All procedures with animals were performed in accordance with the Association for Research in Vision and Ophthalmology (ARVO) statement for use of animals in ophthalmic and vision research and were approved by the institutional Animal Care and Use Committee of the College of Pharmacy, King Saud University.

## Author Contributions

AMA designed the manuscript, supplied funding, interpreted the data, and wrote the manuscript. AA, MN, MS, AD, and LV performed experiments and interpreted the data. PG analyzed the data. GO provided funding, designed experiments, interpreted data, and edited the manuscript. SS provided funding, designed and supervised experiments, interpreted data, and edited the manuscript. All authors read and approved the final manuscript.

## Conflict of Interest

The authors declare that the research was conducted in the absence of any commercial or financial relationships that could be construed as a potential conflict of interest.

## Publisher’s Note

All claims expressed in this article are solely those of the authors and do not necessarily represent those of their affiliated organizations, or those of the publisher, the editors and the reviewers. Any product that may be evaluated in this article, or claim that may be made by its manufacturer, is not guaranteed or endorsed by the publisher.
